# Mebendazole Attenuates Cellular Invasion in a 3D Culture Model of Meningioma by Disrupting Rho-GTPase-Mediated Microtubule Function

**DOI:** 10.32604/or.2026.074958

**Published:** 2026-04-22

**Authors:** Munro Matthew James, López Vásquez Clara Elena, Wickremesekera Agadha, Chan Alex Ho Chuen, Gray Clint Lee

**Affiliations:** 1 Gillies McIndoe Research Institute, P.O. Box 7184, Newtown, Wellington, 6021, New Zealand; 2 Department of Surgery and Anaesthesia, University of Otago, Newtown, Wellington, 6021, New Zealand; 3 Department of Neurosurgery, Wellington Regional Hospital, Wellington, 6021, New Zealand; 4 Department of Paediatrics and Child Health, University of Otago, Newtown, Wellington, 6021, New Zealand; 5 School of Biological Sciences and Centre for Biodiscovery, Victoria University of Wellington, Wellington, 6140, New Zealand

**Keywords:** Meningioma, atypical, meningothelial, mebendazole, invasion assays, proteomics

## Abstract

**Objective:**

Meningioma is the most common primary brain tumour. Invasion into the brain is a diagnostic feature of grade II meningiomas and is associated with recurrence and poor prognosis. Mebendazole is a microtubule inhibitor typically prescribed as an anthelmintic. However, it has the potential to be repurposed for cancer treatment. Here, we aimed to assess the ability of mebendazole to inhibit meningioma cell invasion.

**Methods:**

Primary patient-derived meningioma cell lines were cultured as 3D spheroids and embedded in an extracellular matrix-like matrix as an *in vitro* model of invasion. Mebendazole-treated and untreated control spheroids were analysed by mass spectrometry-based proteomics.

**Results:**

Untreated control spheroids were capable of invasion (9/10 grade I, 10/12 grade II). When treated with mebendazole, invasion was prevented in 89% of samples (8/9 grade I, 9/10 grade II). Mass spectrometry-based proteomics revealed differences between the two grades and between male and female samples within each grade.

**Conclusion:**

Overall, mebendazole reduced meningioma cell invasion via Rho GTPase signalling and altered cytoskeletal dynamics in both male and female patient-derived spheroids. Clearly, more research is needed; however, due to its high tolerability, known safety profile, low cost, and ability to attenuate meningioma cell invasiveness, mebendazole has the potential to be a good candidate for being repurposed for the treatment of meningioma.

## 1 Introduction

Meningioma is the most common tumour of the central nervous system, accounting for more than 35% of such tumours globally [1]. Approximately ~80% of diagnosed meningiomas are Grade I, ~17% are grade II, and <2% are grade III. The differences between grades lie mainly in the mitotic rate and brain invasion rate. Meningiomas are classified as grade I when the mitotic rate is >4 in 10 high-power fields (HPF) and brain invasion does not occur. Grade II presents with 4–19 mitotic figures per 10 HPF, high cellularity, prominent nucleoli, and spontaneous necrosis. Grade III meningiomas present > 20 mitotic figures per 10 HPF and papillary and rhabdoid features [2]. Each grade has several subtypes, with meningothelial being the most common grade I, and atypical for grade II. Meningiomas often cause symptoms depending on their location, such as loss of vision or hearing, dizziness, loss of balance, headache, seizures, and depression. In these cases, standard treatment is surgical removal with or without radiation. However, meningioma cellular invasion can make complete removal of the tumour impossible, limiting the success of surgery. Invasive meningiomas are more likely to be malignant and to recur after initial treatment [3]. Currently, there are no front-line chemotherapeutic options for patients with meningioma [4].

Drug repurposing is the utilisation of an existing drug for a new indication [5]. Repurposed drugs are often off-patent and readily available at lower cost to patients, with proven safety profiles, and should have evidence for penetrating the blood-brain barrier for use in the brain [5,6]. Depending on the way that oral drugs could be applied to disease management, treatment limitations such as proximity to hospitals and socio-economic status can be surmounted. Drug repurposing has the potential to greatly affect access to treatment and, therefore, to more equitable patient outcomes. Mebendazole, an anthelmintic that is typically used to treat worm infections in humans, has demonstrated some anti-cancer evidence of cytotoxicity in cancer cell lines, stimulating antitumoural immune response, reducing metastasis *in vivo*, influencing angiogenesis, pro-survival signalling, matrix metalloproteinases (MMPs) and multidrug resistance transporters, and inhibiting cellular migration and epithelial to mesenchymal transition [7–9]. Furthermore, recent formulation advances for mebendazole have enhanced its solubility and bioavailability, making this a suitable drug candidate for repurposing against meningiomas [10,11]. Mebendazole is a microtubule inhibitor that competitively binds the colchicine-sensitive domain on tubulin, preventing it from polymerising and forming microtubules [12]. It can be administered at relatively high doses (50–70 mg/kg/day), which are well tolerated in adults and children over an extended period (>2 years) with mild side effects that may include bloating, flatulence, abdominal pain, nausea, diarrhoea, headache, tinnitus, and elevated liver enzymes [13]. It has low bioavailability, with <20% reaching circulation, where 90%–95% is protein-bound [7]. Mebendazole does cross the blood-brain barrier (BBB); in mice administered 50 mg/kg/day, a concentration of 7.1 μM was measured in the brain [7]. This suggests that a therapeutically relevant amount of mebendazole can access the brain when administering a typical dosage of mebendazole over an extended period and with only mild side effects. Limited work investigating the utility of mebendazole in the treatment of meningioma has been published [14,15].

Rho GTPases, a subfamily of the Ras superfamily, are small membrane-bound G-proteins. They transduce signals from a wide range of receptors, including cytokine and growth factor receptors such as receptor tyrosine kinases (RTKs), adhesion receptors such as integrins, and G-protein-coupled receptors (GPCRs), in a GTP-dependent manner [16]. The 3 key members of the Rho subfamily are RhoA, Rac1, and Cdc42. Rho GTPases regulate cytoskeletal actin dynamics and cell movement by promoting cellular projections including filopodia (Cdc42), lamellipodia (Rac1), stress fibres, and focal adhesions (upregulated by RhoA; downregulated by Rnd and RhoE) [16,17]. Activation of phosphatidylinositol-3-kinase (PI3K) by GTPases and subsequent phosphorylation of protein kinase B (AKT) upregulates anti-apoptotic signalling and is critical for cell growth, metabolism and cytoskeletal dynamics [18–20].

Three-dimensional (3D) spheroid models have become a more relevant *in vitro* model of representation for tumour physiology and microenvironment as compared to the traditional two-dimensional (2D) monolayer cell cultures [21,22]. Spheroid cultures have cellular interactions with neighbouring cells and the matrix, gradients of nutrients across the spheroid volume, and varying proliferation, quiescent, and hypoxic zones, which can partially replicate the complex microenvironment of tumours [23,24]. Primary cells derived from the tumour that form 3D spheroids offer another advantage, as they retain similarities to the parent tumour [24]. Overall, primary-derived spheroids have a greater physiological representation of meningiomas that would be beneficial for the evaluation of drug responses [22,25].

In the current study, we aimed to determine whether mebendazole could reduce cellular invasion *in vitro* in meningioma spheroids. The mechanisms related to the observed effects on invasion were then investigated by mass spectrometry-based proteomics and pathways analysis, informing on whether mebendazole acted through classical targets or a unique interaction that may have previously been classified as an “off-target effect”.

## 2 Materials and Methods

### 2.1 Establishing Primary Meningioma Cultures

Meningioma tissue samples of varying sizes and weights (Table 1) were obtained according to neurosurgeons’ criteria this study was conducted using consented human tissue samples collected from Wellington Regional Hospital with patients being recruited prior to surgery with informed consent. Approval by Health and Disability Ethics Committee (HDEC, Ministry of Health, New Zealand) was obtained with reference number 15CEN 28. The studies conducted also followed the regulations established in the Declaration of Helsinki. Covered under all necessary ethical HDEC approvals. Tissue samples were immediately transported to Gillies McIndoe Research Institute and processed in two ways: Formalin-Fixation and Paraffin-Embedding (FFPE) and digestion for cell culture mechanically and enzymatically (37°C for up to an hour; collagenase IV 1 mg/mL [ScimaR, cat# LS004188, Brighton, Victoria, Australia], hyaluronidase 0.67 mg/mL [ScimaR, cat# LS002592] and DNase I 0.2 mg/mL [ScimaR, cat# LS002139]) in 10 mL of DMEM (1X) + GlutaMAX-1 (Thermo Fisher, cat# 10569010, Waltham, MA, USA) and 100 μL of Anti-Anti (Antibiotic-Antimycotic (100X), Thermo Fisher, cat# 15240-062). The enzymatic digestion was terminated by adding 1% foetal bovine serum (FBS) (Thermo Fisher, cat# 10091148) to the total volume.

**Table 1:** Background of patient samples used in this study

Sample ID	Subtype	Sex	Age	Ethnicity
18-198	Meningothelial	Female	41	Māori
20-179	Meningothelial	Female	61	European
21-176	Meningothelial	Female	45	Māori
22-093	Meningothelial	Female	66	European
23-003	Meningothelial	Female	63	European
23-019	Meningothelial	Female	66	Asian
18-011	Meningothelial	Male	81	European
19-280	Meningothelial	Male	41	European
20-172	Meningothelial	Male	26	European
23-013	Meningothelial	Male	32	African
23-051	Meningothelial	Male	57	N/A
23-056	Meningothelial	Male	71	N/A
16-050	Atypical	Female	50	Māori
18-349	Atypical	Female	36	European
21-018	Atypical	Female	45	Māori
22-016	Atypical	Female	78	European
23-071	Atypical	Female	52	N/A
17-115	Atypical	Male	61	Māori
18-233	Atypical	Male	24	European
18-258	Atypical	Male	75	European
18-309	Atypical	Male	63	European
18-353	Atypical	Male	79	European
23-014	Atypical	Male	70	European
23-032	Atypical	Male	52	European

Note: Sample ID, subtype, sex, age are ethnicity of all patient samples used in this study.

To isolate single cells, the digest was passed sequentially through 100 and 40 μm filters, with a red blood cell lysis step in between at room temperature for 10 min (155 mM ammonium chloride (Sigma Aldrich, cat# A9434-500G, St. Louis, MO, USA), 12 mM sodium bicarbonate (Merck, cat# 106329.0500, Darmstadt, Germany), 0.1 mM EDTA (Thermo Fisher, cat# 17892)). Around 1 million cells were seeded in laminin-coated (StemCell Technologies, cat# 200-0117, Vancouver, BC, Canada) 6-well plates (Interlab, cat# TCP-010-006, Wellington, New Zealand)(Sapphire Bioscience, cat# LN521-05, Redfern, NSW, Australia) and “Brain Medium” comprising equal parts Advanced DMEM/F-12 (Thermo Fisher, cat# 12634010) and Neurobasal medium (Thermo Fisher, cat# 21103049), with 1× B27 serum replacement without Vitamin A (Thermo Fisher, cat# 12587001), 1× N-2 supplement (Thermo Fisher, cat# 17502001), 1× GlutaMAX supplement (Thermo Fisher, cat# 35050061), insulin (2.5 μg/mL; Sigma Aldrich, cat# I9278-5ML), heparin (2 μg/mL; Tocris, cat# RDS2812100, Bristol, UK), epidermal growth factor (EGF) (25 ng/mL; Lonza, cat# AF-100-15-100UG, Muenchensteinerstrasse, Basel, Switzerland), fibroblast growth factor 2 (FGF2) (20 ng/mL; Lonza, cat# 100-18B-100UG), LIF (10 ng/mL; Sigma Aldrich, cat# SRP3316-25UG), 4% FBS and 1× of Anti-Anti. Mycoplasma testing was done in all patient primary cell lines (Mycoalert Detection Kit—100 tests, Lonza, cat# LT07-318).

To expand cells for future experiments, cells were passaged to T25 (Thermo Fisher, cat# 156367) and T75 (Thermo Fisher, cat#156499) sequentially. Flasks with cells seeded were washed with phosphate-buffered saline (PBS) (Thermo Fisher, cat# 70013032), and TRYPLE (Thermo Fisher, cat# 12605093) was added for 5 min, and the flask was placed inside the incubator at 37°C to lift the cells up. After cells were lifted, more PBS was added to dilute the TRYPLE. Cells collected in Falcon tubes (Interlab, cat# 546021) were then centrifuged, a cell count was performed, and cells were seeded in new coated flasks.

### 2.2 Spheroid Invasion Assay

Primary cell lines were established from a total of 22 meningioma samples—10 grade I meningothelial (5 male, 5 female) and 12 grade II atypical (6 male, 6 female). Cells were seeded at 2.5 × 10^4^ per well in an ultra-low attachment U-bottom 96-well plate (Sigma Aldrich, cat# CLS7007). The plate was centrifuged at 200× *g* for 3 min at room temperature and placed on an orbital shaker in a 37°C incubator for 24 h. Mebendazole (AK Scientific, cat# J10820, San Francisco, CA, USA) was administered at concentrations of μM, 2.5, and 5 μM from a stock solution prepared as per the manufacturer’s instructions. Spheroids were embedded 24 h after the first dose of mebendazole by removing the media and dispensing 30 μL of Cultrex Basement Membrane Extracts (BME) with a protein concentration of 8–12 mg/mL (R&D Systems, cat# 3432-005-01, Minneapolis, MN, USA) into each well using frozen P200 pipette tips and working on ice. The plate was centrifuged at 200× *g* at 4°C for 3 min and placed in the incubator for 30 min to set. Triplicate spheroids for each condition/patient sample were imaged, and drug doses were administered every 24 h for a total of 4 days (T = 0-, 24-, 48-, 72- & 96-h post-embedding) at 4× magnification using the Incucyte SX1 automated live cell analysis instrument (Sartorius, Göttingen, Germany). Our method was adapted from van de Weijer et al. [23].

### 2.3 Embedding Spheroids for Histology

Spheroids were formed by seeding 2 × 10^5^ cells per mL in ultra-low attachment 6-well plates (Sigma Aldrich, cat# CLS3471-24EA), placing the plates on the orbital shaker in the incubator, and allowing spheroids to form over the course of 3–7 days, with a half media change every second day. For histological analysis, individual spheroids were collected using wide-bore P1000 pipette tips, washed with PBS, and fixed with 1 mL of 10% formalin for 20 min at room temperature. Fixed spheroids were washed once with PBS and resuspended in ~100 μL of 2%–3% liquid agarose (55°C) and dispensed into a half-dome mould made using parafilm, which set within 5 min at room temperature. Once set, the agarose domes were placed in a histology cassette and immersed in 10% formalin for 1–2 h to fix the agarose, then transferred to 70% ethanol until being run on a Leica tissue processor ASP200S (Leica, cat# 14 0480 80101, Wetzlar, Germany).

### 2.4 Immunohistochemistry

4 μm FFPE sections of tissues and cultured 3D spheroids underwent haematoxylin and eosin (H&E) staining using Leica Auto stainer XL ST5010 (Leica cat# 14 0456 8010). Once the slides were introduced into the auto stainer, they were baked in the oven for 10 min, followed by two 2 min washes with xylene (TMK Packers, cat# X4, Auckland, New Zealand), two 2 min washes with 99% ethanol (TMK Packers, cat# SDA3A4), 2 min wash station. After this, the slides are submerged in haematoxylin (Milton Adams, cat# G3-1L, Auckland, New Zealand) for 7 mins, washed for 1.5 min, then dipped in acid alcohol (Milton Adams, cat# ACDL-5L) for 9 min, and then submerged in eosin (Milton Adams, cat # EOA1-5L) for 2 min. The slides are then washed again with water for 3 min, dunked twice in 99% alcohol for 2 min, and twice in xylene for 2 min. Slides were checked by a clinical pathologist by comparing the architecture and cellular features of spheroids to their matched parental tissues.

Tissue and spheroid sections were also stained using the Leica Bond RX auto stainer for the meningioma markers epithelial membrane antigen (EMA; Leica, cat# PA0035; ready-to-use) following the immunohistochemistry protocol F from the Leica Bond RX with a heat-induced epitope retrieval (HIER) of 20 min, epitope retrieval 1 (ER1). Somatostatin receptor 2A (SSTR2A; Abcam, cat# ab134252, Cambridge, UK; 1:150) with a 3,3^′^-diaminobenzidine (DAB) step of 30 min, HIER 30 min, ER1. This was done to ensure that cells in culture retained the expression profile of their parental tissue. Staining was validated using positive and negative control tissues and studied by our clinical pathologist to determine the presence or absence of real antibody stain.

### 2.5 Sample Preparation for Mass Spectrometry

Spheroids were formed as above 24 h after seeding cells in the ultra-low adherence plate and placed in shaker overnight. 5 μM mebendazole was added to each well, as well as timepoints T-24-, 48- & 72-h, and spheroids were harvested at T = 96 h to be snap-frozen as dry pellets and stored at −80°C until protein extraction for mass spectrometry.

Sample preparation for mass spectrometry was performed using S-Trap micro columns (ProtiFii, cat# CO2-micro-80, New York, NY, USA) according to the manufacturer’s instructions. Lysis buffer solution was prepared as a 2× stock (10% SDS, 100 mM TEAB, Halt™ Protease and Phosphatase Inhibitor Cocktail [Thermo Fisher, cat# 78440]), and 30 μL were added to spheroid pellets at 1× concentration. Each sample contained between 24–30 spheroids. The pellet was mechanically disrupted using a P200 pipette and benchtop vortex, then placed in a sonic water bath for 2 × 2 min before agitating on an orbital shaker for 40 min at 350 rpm. Tubes were then briefly vortexed and centrifuged at 13,000× *g* at 4°C for 8 min. Supernatant was retained in a fresh labelled tube. Protein concentration was measured by Pierce^TM^ BCA assay (Thermo Fisher, cat# 23227). Up to 100 μg of protein lysate in 23 μL was taken, and 1 μL of 0.5 M TCEP (Thermo Fisher, cat# 77720) was added, and the tubes were placed in a heat block on an orbital shaker at 95°C for 10 min. Fresh stock of 400 mM iodoacetamide (Sigma Aldrich, cat# A3221-10VL) was prepared, and 3 μL was added to each tube and incubated in the dark at room temperature for 30 min. Next, 3 μL of 12% phosphoric acid and 180 μL of S-Trap binding buffer (90% methanol, 100 mM TEAB [Sigma Aldrich, cat#T7408]) were added to each tube and briefly vortexed. Proteins were captured on S-Trap micro columns and washed with 100 μL S-Trap binding buffer at 13,000× *g* for 1 min at room temperature for 4 times. Captured proteins were digested with 2 μg trypsin (1:20–1:50 trypsin-to-protein ratio) (Promega, cat# PMV5111, Madison, WI, USA) in a humidified chamber at 37°C for 16 h. To elute the digested peptides from the column, three sequential elutions using 50 mM TEAB, 0.2% formic acid, and 50% acetonitrile + 0.2% formic acid were performed. The eluted peptides were lyophilised and stored at −20°C until proceeding to the next step. Lyophilised peptide samples were reconstituted, and concentrations were measured again by Pierce^TM^ BCA assay. Samples were diluted in reconstitution buffer (2% acetonitrile (Merck, cat# 1000301000) and 0.05% trifluoroacetic acid (Sigma Aldrich, cat# T6508-5ML) in MilliQ water) to 0.5 μg/μL and transferred to proteomic autosampler vials and stored at −80°C. Samples were sent to the Mass Spectrometry and Proteomics Facility in Bio21 Institute, University of Melbourne, using the Thermo Orbitrap Astral Mass Spectrometer for data-independent acquisition (DIA).

### 2.6 Mass Spectrometry Data Analysis

The human proteome (UniProt: https://www.uniprot.org/) was uploaded to DIA-NN Software to generate a spectral library based on experimental data to identify proteins from the raw peptide-spectrum matches (PSMs) [24]. Raw data was filtered at a 1% false discovery rate. Raw data was filtered at 0.01 FDR in DIA-NN, prior to further analysis with Perseus and Ingenuity Pathway Analysis Software 2024. Identified proteins were uploaded to Perseus Software to analyse coverage, quality assessment, protein annotations, data transformation, data normalisation, and data visualisation quality assessment, protein annotations, data transformation, data normalisation, and data visualisation [25]. Data was filtered such that identified proteins were only retained if there were valid quantification data across at least 80% of the samples. The filtered dataset underwent a log_2_(x) transformation. Any values that were missing from the dataset at this stage were then imputed from the normal distribution and normalised by subtracting the most frequent value within columns. Data was then annotated with Gene Ontology (GO) terms and Kyoto encyclopaedia of genes and genomes (KEGG) pathways using the UniProt reference database. Annotations included protein name, protein group, protein description, protein function, protein location, and genes. The following data for each comparison (treated vs. untreated; male vs. female; grade I vs. grade II) that was statistically analysed using Student’s *t*-test, was then exported from Perseus and uploaded to Ingenuity Pathway Analysis Software 2024 (IPA; QIAGEN, Hilden, Germany): *p*-value, fold change, and UniProt protein identifiers (accession numbers). Using IPA, setting a fold change cut-off of 2.5, canonical pathways enriched in each group were identified, networks consisting of interacting pathways were created and interrogated [26].

### 2.7 Statistical Analysis

Two-way (concentration × time) ANOVA (with repeated measures when necessary) using Dunnett’s multiple comparison test was performed in GraphPad Prism 9 (GraphPad Software, Inc., San Diego, CA, USA) to compare mebendazole-treated groups to untreated controls and assess the statistical significance of altered invasion. On demonstrating a non-normal distribution or heteroscedasticity, we applied log10 as appropriate. All analyses were repeated using the transformed data, and results were interpreted using these corrected models unless otherwise stated. Data presented as mean ± SEM. Two-sample *t*-tests were performed in IPA for proteomics data group comparisons. A significant difference was determined when *p* < 0.05.

## 3 Results

### 3.1 Primary Spheroids Express Markers Similar to Parental Tissues

**Figure 1:**
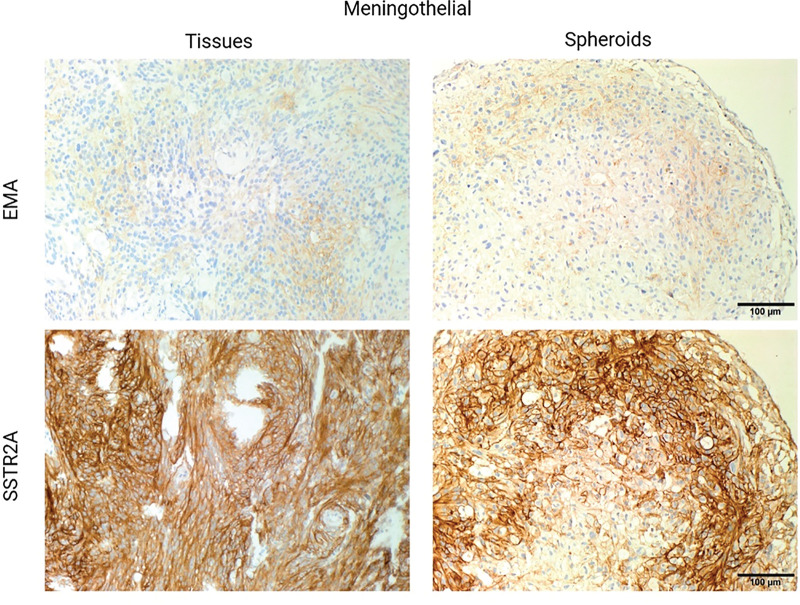

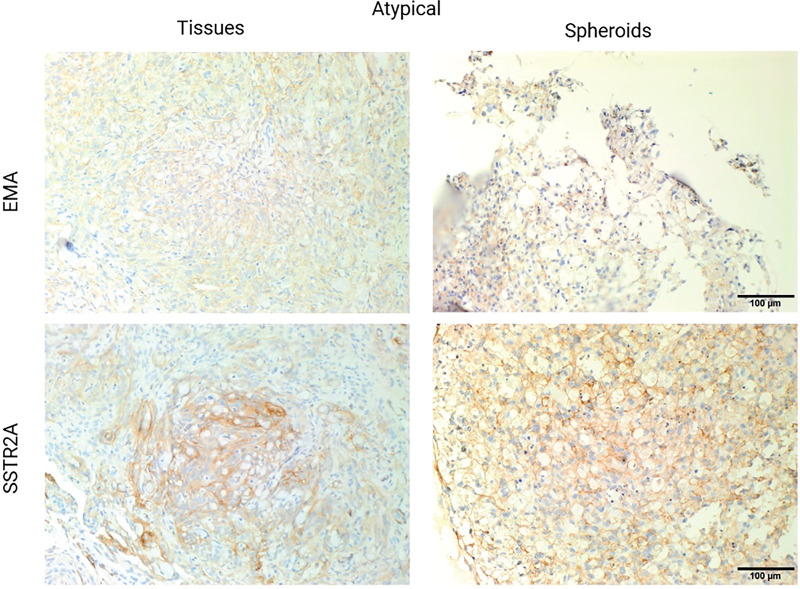
Representative immunohistochemistry (IHC) images of patient-derived meningioma spheroids and their patient-matched parental tissues. Immunohistochemical staining of meningioma markers epithelial membrane antigen (EMA) and somatostatin receptor 2A (SSTR2A) in representative meningothelial (top) and atypical (bottom) tissues (left) and patient-matched spheroids (right). Stains were analysed by our pathologist to confirm the presence or absence of the marker. Magnification = 20×

The expression of epithelial membrane antigen (EMA) and somatostatin receptor 2A (SSTR2A), two common diagnostic biomarkers of meningioma, in the tissues matched the expression in spheroids of meningothelial and atypical samples. This was assessed through immunohistochemical staining and confirmed by our pathologist that the patterns of staining intensity and distribution seen in the parental tissues are recapitulated in the spheroids (Fig. 1).

It was expected for SSTR2A to be present in the cytoplasm and membrane, as shown in both parental tissues and spheroids. According to the literature, this protein results in a better biomarker because of its abundance in meningioma [27,28]. Despite EMA being present with a weak stain in the cytoplasm, it showed the same distribution and intensity in both compared groups, tissue and spheroids, as well as what has been mentioned previously in the literature [27,28].

### 3.2 Mebendazole Reduces the Invasive Capability of Meningioma Spheroids in a Dose-Dependent Manner

**Figure 2:**
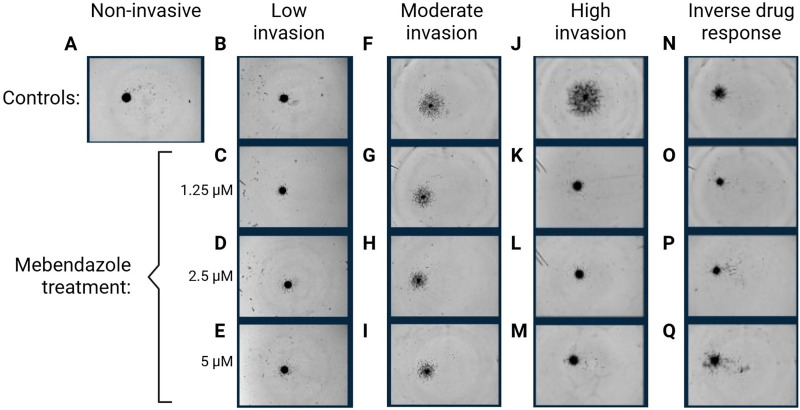
Effect of mebendazole treatment on the invasive capacity of meningioma spheroids. Representative images after 96 h from meningioma invasion assays, showing examples of samples with no invasion in the untreated control (**A**); low basal invasion (**B**) with drug response (**C**–**E**); moderate basal invasion (**F**) with modest drug response (**G**–**I**); and high basal invasion (**J**) with strong drug response (**K**–**M**). Also shown is a sample that exhibited an unusual drug response, with invasion increasing at the highest dose of mebendazole (**N**–**Q**)

Fig. 2A shows an embedded spheroid that did not invade. This shows that not all patient spheroids have a basal invasion. This patient was removed from the analysis. Fig. 2B–E shows one of the three patient samples that presented low levels of invasion in control and 1.25, 2.5, and 5 μM of mebendazole treatment. Fig. 2F–I shows one of the six patient samples that presented moderate levels of invasion in control and 1.25, 2.5, and 5 μM of mebendazole treatment. Fig. 2J–M shows one of the patient samples that presented high levels of invasion in control, and this invasion was reduced when treated with 1.25, 2.5, and 5 μM of mebendazole. Fig. 2N–Q shows one patient sample that presented inverse drug response at all concentrations, increasing invasion at higher concentrations of mebendazole. One sample with low basal invasion displayed reduced invasion at low dose (−40% at 1.25 μM) but enhanced invasion at higher doses of mebendazole (+23% at 2.5 μM and +87% at 5 μM) (Fig. 2C–E). The two other samples with low basal invasion saw reduced invasion (>30% at 1.5, 2.5 & 5 μM) when treated with all three doses of mebendazole. When mebendazole was able to reduce invasion, the samples tended to be either moderate (50%–75% reduction; Fig. 2G–I) or high responders (>80% reduction; Fig. 2K–M) across all three doses. Collectively, a dose-dependent effect was observed as increasing concentrations of mebendazole reduced the invasion of meningothelial spheroids at progressively earlier timepoints; immediately after embedding with 5 μM (*p* ≤ 0.0104); from 48 h post-embedding with 2.5 μM (*p* ≤ 0.0128); and from 72 h post-embedding with 1.25 μM (*p* ≤ 0.0268).

The effect of mebendazole on invasion was more variable in the atypical spheroids than in meningothelial spheroids. Two out of the twelve atypical-derived spheroids failed to invade (Fig. 2J). Two samples had a positive dose response trend, displaying greater inhibition of invasion with increased dose, while another sample had the opposite dose response trend with reductions of 87% and 34% at 1.25 and 2.5 μM, respectively, but an increase of invasion of 38% at 5 μM (Fig. 2N–Q). Six samples displayed a reduction in invasion greater than 85% at all three doses, including one of the highly invasive samples; the other highly invasive spheroid saw reductions of 71%–83% across doses. Similarly, a dose-dependent effect of mebendazole was observed on atypical spheroids to reduce invasion at progressively earlier timepoints; immediately after embedding with 5 μM (*p* ≤ 0.0089); and from 48 h post-embedding with 2.5 μM (*p* ≤ 0.0271) and 1.25 μM (*p* ≤ 0.0446).

### 3.3 Invasion of Mebendazole-Treated Meningioma Spheroids Versus Untreated Controls

**Figure 3:**
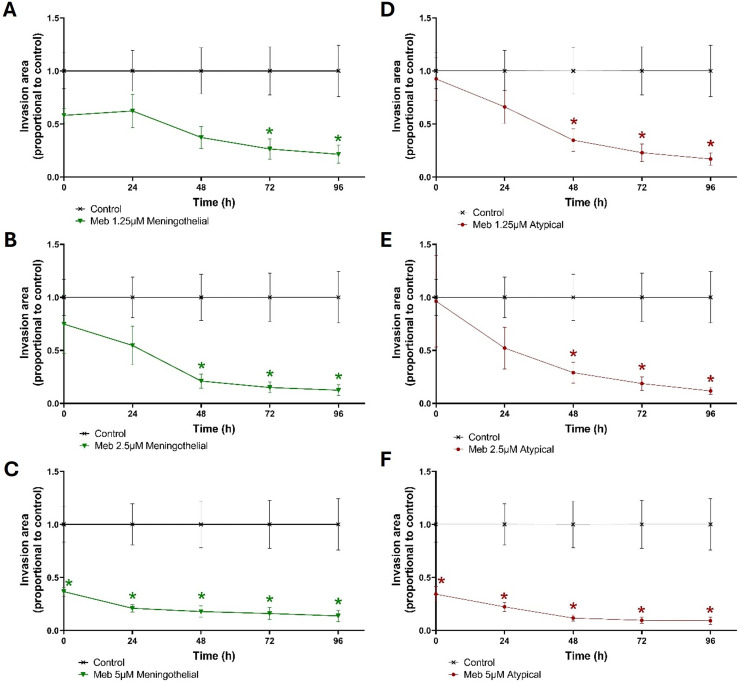
Invasion of mebendazole-treated meningioma spheroids vs. untreated controls. Invasion of meningothelial (left) and atypical (right) meningioma spheroids treated with mebendazole at doses of 1.25, 2.5, or 5 μM, proportional to untreated control spheroids over the course of 96 h. Drug was administered at T = −24, 0, 24, 48 & 72 h from embedding. Meningothelial n = 9, atypical n = 10, error bars = SEM; *indicates a significant difference of *p* < 0.05 when compared to the vehicle controls, *p < 0.05

Mebendazole showed a significantly reduced invasion in eight of the nine meningothelial invasive samples and 9 of the 10 atypical spheroid samples (Fig. 3). Fig. 3A–C shows the invasion area of meningothelial spheroids at three different mebendazole concentrations (1.25, 2.5, or 5 μM) in relation to untreated controls. Fig. 3D–F also reduced invasion in atypical meningioma spheroids. In both subtypes, the level of invasion was reduced significantly at higher doses (*p < 0.05) and in longer treatment periods, being significant in all concentrations at 72 and 96 h (*p < 0.05).

### 3.4 Pathways Differential Expression in Atypical Spheroids Relative to Meningothelial Spheroids

**Figure 4:**
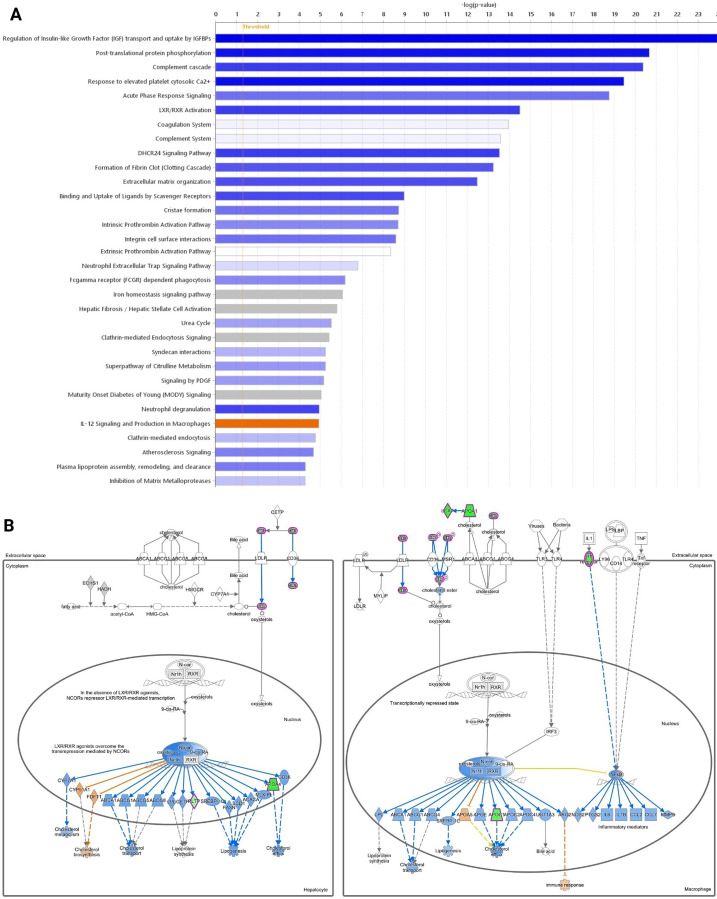
Pathways differential expression in atypical spheroids relative to meningothelial spheroids. (**A**) Comparison between untreated atypical vs. meningothelial spheroids. Pathways that have statistical significance of *p* < 0.05 with activity levels that are predicted to be downregulated (blue) or upregulated (orange), altered without change in the overall activity levels (white), or dysregulated without enough data to infer the direction of change (grey) in atypical relative to meningothelial are shown. (**B**) Members of the LXR/RXR pathway with expression measured (green) or predicted (blue) to be lower, or predicted to be higher (orange), in atypical spheroids relative to meningothelial are shown

Spheroids derived from untreated meningothelial and atypical meningiomas were compared to identify the underlying differences between the two grades. Relative to meningothelial spheroids, the atypical spheroids were enriched for IL-12 signalling (*p* = 1.2 × 10^−5^) (Fig. 4A, orange). Conversely, the meningothelial spheroids were enriched for immune responses, insulin-like growth factor (IGF) transport (*p* = 1.2 × 10^−24^), extracellular matrix organisation (*p* = 3.4 × 10^−13^), DHCR24 signalling in cholesterol biosynthesis (*p* = 2.9 × 10^−14^) (Fig. 4A, blue), and LXR/RXR signalling in lipid synthesis and transport (*p* = 3.14 × 10^−15^; Fig. 4B).

### 3.5 Comparison between Male and Female Meningioma Spheroids

**Figure 5:**
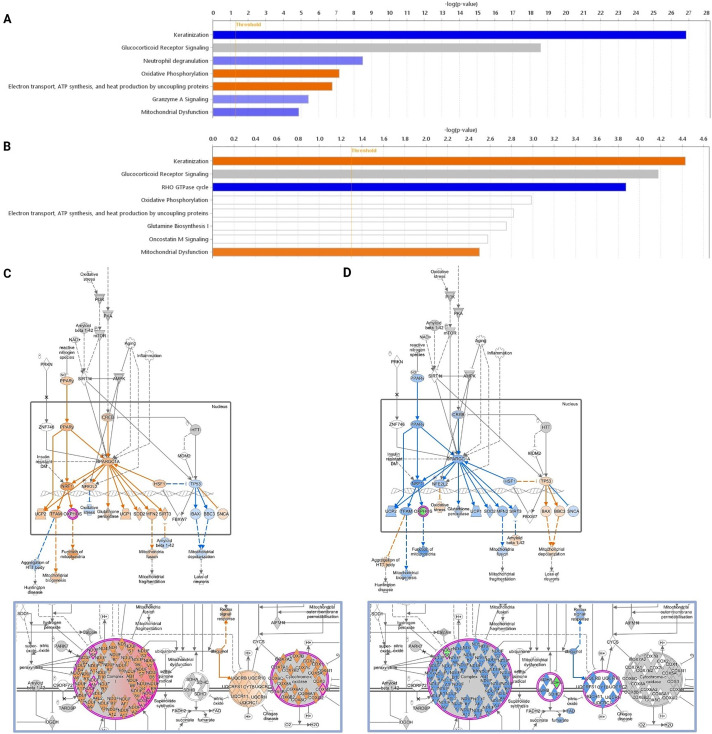
Comparison between male and female meningioma spheroids. Comparison of meningothelial male vs. female spheroids (**A**), and atypical male vs. female spheroids (**B**). Pathways that have statistical significance of *p* < 0.05 with activity levels that are lower (blue) or higher (orange), have differential abundance or activity of individual components without overall changes to pathway activity (white), or are dysregulated without enough data to infer the direction of change (grey) are shown. In male vs. female untreated meningothelial spheroids, the “mitochondrial dysfunction” and “OXPHOS” pathways were predicted to be downregulated and upregulated, respectively (**C**), but the opposite was predicted in untreated atypical spheroids (**D**)

Male and female untreated control samples within each grade were then compared. Spheroids derived from male meningothelial samples had higher OXPHOS (*p* = 6.8 × 10^−8^), electron transport, ATP synthesis and response to oxidative stress (*p* = 1.7 × 10^−7^) (Fig. 5A, orange), whereas female meningothelial spheroids had higher keratinisation (*p* = 1.4 × 10^−27^), neutrophil degranulation (*p* = 3.2 × 10^−9^), mitochondrial dysfunction (*p* = 1.4 × 10^−5^) and granzyme A signalling (*p* = 3.8 × 10^−6^) (Fig. 5A, blue). In contrast, the male atypical spheroids had increased mitochondrial dysfunction (*p* = 0.003) and keratinisation (*p* = 3.7 × 10^−5^) (Fig. 5B, orange), and the female atypical spheroids for the Rho GTPase cycle (*p* = 1.3 × 10^−4^) (Fig. 5B, blue). Of note was the disparity between grades in terms of mitochondrial dysfunction, where the meningothelial males displayed greater levels of OXPHOS than the females, which had greater mitochondrial dysregulation (Fig. 5C), whereas the opposite was observed in the atypical spheroids (Fig. 5D).

### 3.6 Effects of Mebendazole on Meningothelial Meningioma Spheroids

**Figure 6:**
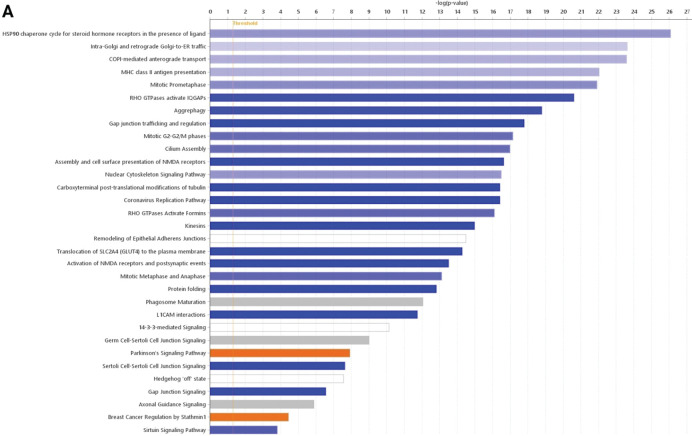

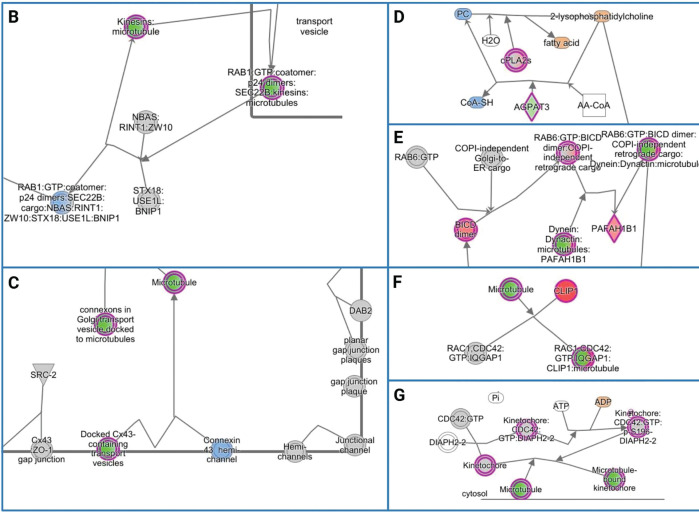
Effects of mebendazole on meningothelial meningioma spheroids. The effect of mebendazole treatment on canonical pathways in meningothelial meningioma spheroids (**A**). Pathways that have statistical significance of *p* < 0.05 with activity that was downregulated (blue), upregulated (orange), altered without change in the overall activity levels (white), or dysregulated without enough data to infer the direction of change (grey) in mebendazole-treated meningothelial spheroids relative to untreated controls. Examples of proteins with altered activity or abundance in response to mebendazole in gap junction signalling (**B**,**C**), Golgi transport (**D**,**E**), and Rho signalling (**F**,**G**); green = measured reduction, blue = predicted reduction, red = measured increase, orange = predicted increase

As observed through the invasion assays, this feature in meningothelial meningioma spheroids is attenuated by mebendazole treatment. However, there were some differences in terms of the pathways inhibited by mebendazole in meningioma spheroids. Meningothelial spheroids displayed a reduction in Rho GTPase activity, particularly their activation of downstream effectors, including formins, IQGAP scaffold proteins, WASPs, WAVEs and p21-activated serine/threonine kinases (PAKs), as well as Golgi trafficking. The meningothelial spheroids showed a reduction in proteins involved in a number of other pathways, predominantly microtubule-associated functions such as gap junctions (*p* = 1.6 × 10^−18^), the nuclear cytoskeleton (*p* = 3.2 × 10^−17^), antigen presentation by MHC class II (*p* = 9.3 × 10^−23^), cilium assembly (*p* = 1 × 10^−17^), and tubulin post-translational modification (*p* = 3.8 × 10^−17^), as well as protein folding mediated by the heat shock protein (HSP90) chaperone (*p* = 8.3 × 10^−27^) (Fig. 6A). Spheroids derived from meningothelial samples displayed reduced microtubule-related functions such as trafficking and signalling from gap junctions (Fig. 6B,C) and the Golgi (Fig. 6D,E), Rho GTPase activity (Fig. 6F,G), and protein folding (Fig. 6A).

### 3.7 Comparing the Response of Male and Female Meningothelial Spheroids to Mebendazole Treatment

**Figure 7:**
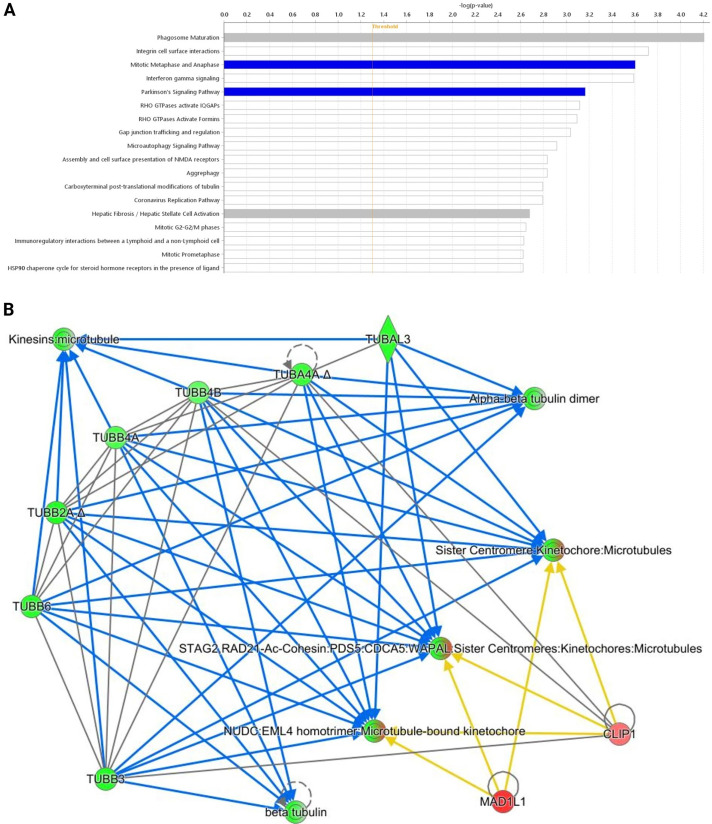
Comparing the response of male and female meningothelial spheroids to mebendazole treatment. (**A**) Comparison between mebendazole-treated male vs. female meningothelial spheroids. Pathways that have statistical significance of *p* < 0.05 with activity levels that are lower (blue), altered without change in the overall activity levels (white) or altered without enough data to infer direction of change (grey) in male relative to female after mebendazole treatment are shown. (**B**) An interaction network showing selected proteins involved in microtubule dynamics in meningothelial meningioma spheroids; altered activity or abundance of protein nodes in response to mebendazole shown as: green = measured reduction, blue = predicted reduction, red = measured increase, orange = predicted increase

Within meningothelial samples, very similar responses to mebendazole were seen in males and females. Male spheroids showed a downregulation in the mitotic metaphase and anaphase pathway as well as Parkinson’s signalling pathway (Fig. 7A). They displayed reduced tubulin post-translational modification, aggrephagy, cilium formation, antigen presentation by MHC Class II, glucose transporter 4 (GLUT4) translocation, and nuclear cytoskeleton dynamics (Fig. 7B).

### 3.8 Effects of Mebendazole Treatment on Atypical Meningioma Spheroids

**Figure 8:**
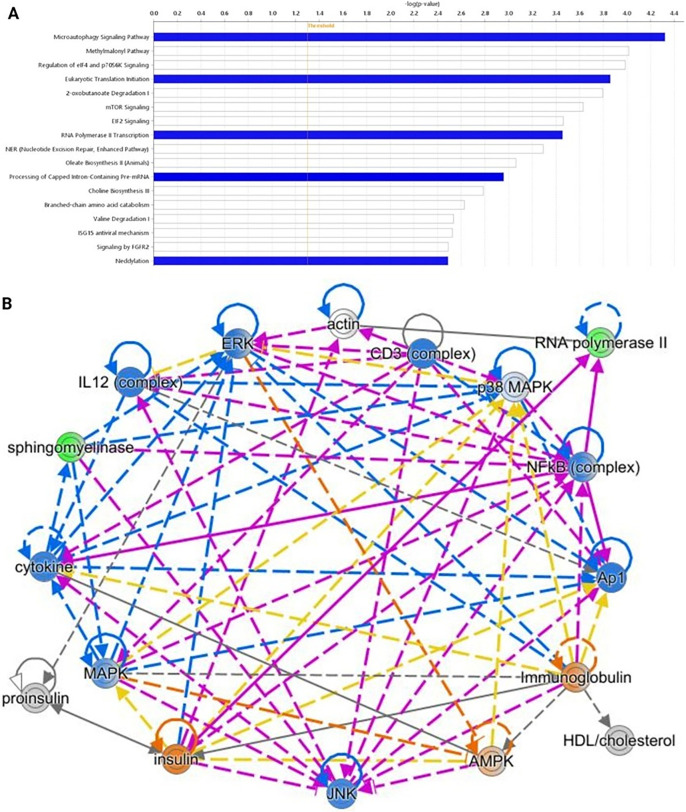
Effects of mebendazole treatment on atypical meningioma spheroids. **(A**) Comparison between mebendazole-treated and untreated control atypical spheroids; pathways that have statistical significance of *p* < 0.05 with activity levels that are lower (blue) or altered without change in the overall activity levels (white) in mebendazole-treated relative to untreated controls are shown. (**B**) An interaction network showing selected proteins involved in signalling in atypical spheroids; altered activity or abundance in response to mebendazole shown as: green = measured reduction, blue = predicted reduction, red = measured increase, orange = predicted increase. Solid arrows = direct interaction, dashed arrows = indirect interaction

Mebendazole-treated atypical spheroids had reduced mTOR-induced transcription and translation (*p* = 2.4 × 10^−4^), altered FGFR2 signalling (*p* = 0.003), impaired microautophagy (*p* = 4.8 × 10^−5^), and decreased protein neddylation (*p* = 0.003) (Fig. 8A). Cellular proliferation and signalling mediators such as EGFR, FGFR2, JNK, ERK, MAPK, insulin and NF-kB, which are more heavily implicated in grade II meningioma than grade I, were attenuated by mebendazole to a larger degree in the atypical (grade II) spheroids than the meningothelial (grade I) spheroids (Fig. 8B).

Overall, mebendazole affected meningioma spheroids across grades and sexes by disrupting microtubule function, which is influenced by Rho GTPase signalling and is implicated in a myriad of cellular functions, including intracellular trafficking, cellular migration, and invasion.

## 4 Discussion

Our study has utilised patient-derived primary meningioma spheroid cultures to perform an untargeted mass spectrometry-based proteomic analysis following pre- and post-treatment with mebendazole. Before treatment, relative to meningothelial spheroids, atypical spheroids displayed lower synthesis and transport of lipids and cholesterol, extracellular matrix features, mitochondrial metabolism, IGF signalling, and complement and coagulation cascade activity, similar to previous reports [29–31]. Following treatment with mebendazole, atypical spheroids displayed downregulated RNA metabolism and processing and chromatin remodelling, features previously attributed to atypical meningioma [29]. Our finding that mebendazole treatment downregulated HSP90 activity specifically in meningothelial spheroids, the most common subtype of grade I meningiomas, further aligns with reports of HSP90 activators being more highly expressed in grade I meningiomas than grade II meningiomas [31]. Our comparison of grade I and grade II meningiomas using proteomics echoed the results of earlier studies and additionally found that certain features associated with meningioma subtypes can be attenuated by mebendazole treatment. Differences between our study and those cited above could be due to our focus on the meningothelial subtype, rather than investigating all grade I subtypes collectively.

We hypothesised that mebendazole, a microtubule inhibitor, would reduce the invasive capacity of meningioma cells. In the current study, mebendazole reduced and prevented, in part, invasion in 8 out of 9 invasive meningothelial spheroids and 9 out of 10 invasive atypical meningioma spheroids (89.5%) at a dose as low as 1.25 μM. The estimated 1.25 μM brain concentration is extrapolated from murine pharmacokinetic data and remains speculative for humans. Dedicated clinical pharmacokinetic studies are needed to determine whether comparable concentrations can be achieved [7]. Two pathways were significantly downregulated following mebendazole treatment were the activation of (1) formins and (2) IQGAPs by Rho GTPases, suggesting that mebendazole may interfere with meningioma cell invasion by attenuating Rho GTPase activity. This has downstream effects on cell morphology and polarity (e.g., for mitosis); cell adhesion, movement, and migration via extensions, contractions, and regulation of adherens junctions and tight junctions; wound healing, including axonal (re)generation; and vesicular trafficking, involved in exocytosis, endocytosis, and phagocytosis [16,32]. Similarly, mebendazole-treated meningothelial spheroids were also observed to downregulate receptor localisation to membranes, gap junction trafficking and signalling, and adherens junction remodelling, further revealing the potential impact of mebendazole-induced alterations to microtubule dynamics in meningioma cells. Rho GTPases contribute to the cell cycle, mitosis, and cell division. They promote G1-S progression by repressing cyclin-dependent kinase inhibitors and inducing cyclin D1 [33]. Previous reports have correlated cell cycle and mitosis upregulation with higher meningioma grade [34]. Here, we report that all phases of mitosis were downregulated by mebendazole treatment, particularly in meningothelial spheroids, presumably through the blockade of microtubule formation in the nucleus. Furthermore, mebendazole downregulated aggrephagy, microautophagy, intra-Golgi trafficking, Golgi-to-ER retrograde trafficking, and trans-Golgi vesicle budding, implicating mebendazole in disrupting other processes directed by Rho GTPase signalling. These processes have previously been identified as features of meningioma [29,30]. Collectively, this suggests that the perturbation of Rho GTPase signalling is the predominant mechanism through which mebendazole prevents meningioma cell invasion.

Interestingly, the uptake and transport of IGF were significantly dysregulated in atypical meningioma spheroids relative to meningothelial cells. The phosphatidylinositol-4,5-bisphosphate 3-kinase catalytic subunit alpha (*PIK3CA)* mutation, common in both meningothelial and atypical meningiomas, leads to constitutive activation of PI3K signalling downstream of RTKs and upstream of AKT [35]. PIK3CA acts as a signal transducer between RTKs and Rho GTPases to facilitate vesicle trafficking [19]. Signalling via insulin-like growth factor receptor (IGF1R), an RTK, activates PI3K to induce AKT recruitment and subsequent promotion of survival, proliferation, and differentiation pathways [36]. Therefore, interference of IGF signalling may, in part, contribute to the anti-invasive activity of mebendazole in meningioma.

The PI3K pathway provides cancer cells with a competitive advantage by modulating metabolism, especially glycolysis. Proteomics revealed that mebendazole reduced the expression of insulin-regulated glucose uptake transporter GLUT4 on the cell membrane in meningothelial spheroids. This may indicate a reduction in glucose cellular uptake. However, without performing any metabolic studies, we are unable to prove this hypothesis in the current study. Cancers, including meningioma, are highly reliant on glycolysis for energy generation via the Warburg effect, and so the reduction of intracellular glucose slows cancer cell metabolism [37]. GLUT4 expression by neural stem cells (NSCs) increases with age as mitochondrial respiration decreases, and this is accompanied by dysregulation of NSC function [38]. RhoA/ROCK signalling may control the transport of GLUT4 to the cell membrane as it does for GLUT1, contributing to the utilisation of the Warburg effect [33]. Selke et al. reported that reduced glucose levels mitigate meningioma cell invasion [37]. Moreover, mebendazole has previously been shown to reduce glucose absorption [7]. Our results suggest that mebendazole may reduce the ability of meningioma cells to import glucose, a potentially vital fuel for their migration, via differential GLUT4 expression.

The interaction between mammalian target of rapamycin complex 2 (mTORC2) and GTPases is a driver of cytoskeletal regulation [39]. Others have shown that GSK3 phosphorylates Rho-GDP, allowing it to form a complex with Ras-GTP and mTORC2 that subsequently phosphorylates AKT [40].

Proteomics data shown here suggest that meningothelial-derived spheroids had higher activity of AKT and NF-κB than atypical spheroids. The E17K hotspot mutation that constitutively activates AKT leads to upregulation of signalling via PI3K/AKT, mTOR, and ERK1/2 [41,42]. This mutation is also responsible for increased inflammatory mediators, including NF-κB, which can be activated by RhoA, Rac1, and Cdc42 to prevent apoptosis, sometimes downstream of integrin signalling [33,43]. Inflammatory cytokines can activate RhoA/NF-κB signalling to induce cellular invasion [33]. Our results align with previously observed enrichment of the E17K mutation in the meningothelial subtype [42].

Reduced GTPase activity as observed in mebendazole-treated meningioma spheroids could help alleviate the effects of the common *TRAF7* mutation in meningioma. TRAF7 is a proapoptotic protein with E3 ubiquitin ligase activity that regulates small GTPases [41]. *TRAF7* loss-of-function mutations, the second-most common mutation in meningiomas, are mutually exclusive with *NF2* mutations and are almost always associated with skull base meningioma [44]. They can occur in meningothelial and atypical subtypes, often co-occurring with the meningioma-specific loss-of-function K409Q mutations in the *KLF4* gene and/or activating mutations to AKT1 and PIK3CA [30]. *TRAF7* mutations lead to upregulation of RAS/MAPK signalling upstream of ERK1/2 and mTORC1 [39,44].

Although meningioma is usually a benign tumour, it can have debilitating symptoms due to the physical pressure it exerts on the brain. Meningiomas that invade the brain are often unable to be completely resected, increasing the risk of progression and recurrence. When treated with mebendazole *in vitro*, the invasive capability of primary patient-derived meningioma spheroids was significantly attenuated. This was the case in ~90% of the meningothelial and atypical samples tested (8 out of 9 meningothelial, 9 out of 10 atypical). Using proteomic analyses, it was determined that mebendazole exerts this effect largely through its ability to disrupt Rho GTPase signalling and microtubule dynamics. Many pathways and processes linked to Rho signalling were altered by mebendazole, including potential glucose uptake by GLUT4, intracellular trafficking, cell junctions, cell cycle and mitosis, and the PI3K/AKT/mTOR and MAPK signalling cascades.

While our findings provide insight into the role of mebendazole in meningiomas, there are several limitations that should be acknowledged. Both a limitation and a strength to the study was using primary cells derived from patient tumours, where interindividual variations will result in different innate behaviours and responses to mebendazole treatment, while also accounting for real-world phenotypes of meningiomas. As a result, three cell lines were excluded due to having no baseline invasion, two cell lines did not respond to treatment, and two cell lines had a paradoxical increase in invasion with a higher dose of mebendazole. The lack of response and paradoxical response from mebendazole may be due to interindividual differences within the tumours. Potential confounding factors that may be responsible for the differential response could be due to tumour age, origin of tumour, or molecular mutations that were not accounted for due to the limited availability of samples. Future studies should delve into the underlying molecular differences between meningiomas of the same grade that could alter the response to the same treatment. Another limitation that should be acknowledged was that the effect of mebendazole was not compared to other existing microtubule inhibitor treatments, which may provide insight into the differential responses from the non-responsive and paradoxical response spheroids.

## 5 Conclusion

Our study shows that mebendazole can reduce the invasion of meningioma cells and offers insight into the mechanisms responsible for this. Therefore, further studies should explore deeper into the mechanisms and pharmacokinetics of mebendazole, as it may be a viable candidate to be repurposed for the future treatment against grade I and II meningiomas.

## Data Availability

The data that support the findings of this study are available from the Corresponding Authors, [Munro Matthew James, Gray Clint Lee], upon reasonable request.
